# Indices of Defective Autophagy in Whole Muscle and Lysosome Enriched Fractions From Aged D2-mdx Mice

**DOI:** 10.3389/fphys.2021.691245

**Published:** 2021-07-09

**Authors:** Swathy Krishna, Hannah R. Spaulding, Tiffany S. Quindry, Matthew B. Hudson, John C. Quindry, Joshua T. Selsby

**Affiliations:** ^1^Department of Animal Science, Iowa State University, Ames, IA, United States; ^2^School of Integrative Physiology and Athletic Training, University of Montana, Missoula, MT, United States; ^3^Department of Kinesiology and Applied Physiology, University of Delaware, Newark, DE, United States

**Keywords:** dystrophin, Duchenne muscular dystrophy, mouse model, diaphragm, gastrocnemius, D2-mdx

## Abstract

Duchenne muscular dystrophy (DMD) is a fatal, progressive muscle disease caused by the absence of functional dystrophin protein. Previous studies in mdx mice, a common DMD model, identified impaired autophagy with lysosomal insufficiency and impaired autophagosomal degradation as consequences of dystrophin deficiency. Thus, we hypothesized that lysosomal abundance would be decreased and degradation of autophagosomes would be impaired in muscles of D2-mdx mice. To test this hypothesis, diaphragm and gastrocnemius muscles from 11 month-old D2-mdx and DBA/2J (healthy) mice were collected. Whole muscle protein from diaphragm and gastrocnemius muscles, and protein from a cytosolic fraction (CF) and a lysosome-enriched fraction (LEF) from gastrocnemius muscles, were isolated and used for western blotting. Initiation of autophagy was not robustly activated in whole muscle protein from diaphragm and gastrocnemius, however, autophagosome formation markers were elevated in dystrophic muscles. Autophagosome degradation was impaired in D2-mdx diaphragms but appeared to be maintained in gastrocnemius muscles. To better understand this muscle-specific distinction, we investigated autophagic signaling in CFs and LEFs from gastrocnemius muscles. Within the LEF we discovered that the degradation of autophagosomes was similar between groups. Further, our data suggest an expanded, though impaired, lysosomal pool in dystrophic muscle. Notably, these data indicate a degree of muscle specificity as well as model specificity with regard to autophagic dysfunction in dystrophic muscles. Stimulation of autophagy in dystrophic muscles may hold promise for DMD patients as a potential therapeutic, however, it will be critical to choose the appropriate model and muscles that most closely recapitulate findings from human patients to further develop these therapeutics.

## Introduction

Duchenne muscular dystrophy (DMD) is a progressive muscle-degenerative disease caused by the absence of functional dystrophin protein (Hoffman et al., 1987). Dystrophin plays an important role in maintaining structural stability by establishing a link between cytoskeletal actin and the extracellular matrix (Petrof et al., 1993b; Ehmsen et al., 2002). Dystrophin deficiency causes disruption of the dystrophin associated protein complex (DAPC) and leads to sarcolemmal fragility particularly during eccentric contractions (Petrof et al., 1993b). The resultant cellular injuries and altered signaling originating from the DAPC (Rahimov and Kunkel, 2013; Houang et al., 2018) initiate a cascade of malfunction in muscle fibers (Selsby et al., 2010, 2012; Selsby, 2011; Spaulding et al., 2018) that ultimately cause injury, inflammation, fibrosis, and fatty infiltration at the whole muscle level (Chen et al., 2005; Morris et al., 2010).

In cells from healthy muscle, alterations to autophagic flux can maintain homeostasis during cell stress or injury (Sandri, 2010; Castets et al., 2016). During autophagy, damaged organelles and proteins are sequestered in autophagosomes and then subjected to lysosomal degradation (reviewed in Parzych and Klionsky, 2014; Farre and Subramani, 2016; Kocaturk and Gozuacik, 2018). We and others have reported defective autophagy and increased autophagosome efflux, termed autophagosome escape, in dystrophin-deficient muscle from mdx mice and the limited data from human DMD patients suggest similar outcomes (De Palma et al., 2012; Pauly et al., 2012; Spitali et al., 2013; Castets et al., 2016; Spaulding et al., 2018, 2020a). These data raise the possibility that stimulation of autophagy or degradation of autophagosomes may be effective therapeutic strategies for DMD (Hollinger and Selsby, 2013; Pal et al., 2015; Spaulding et al., 2020a). Indeed, pharmacological activation of AMP-activated protein kinase (AMPK), a known activator of autophagy, and stimulation of transcription factor EB (TFEB), a transcription factor that drives transcription of autophagic machinery, implicates dysfunctional autophagy as part of the dystrophic disease process, but also demonstrates the potential therapeutic value of increased autophagy for dystrophin deficiency (Ljubicic et al., 2012; Pauly et al., 2012; Spaulding et al., 2020a).

Autophagic degradation is facilitated by lysosomal enzymes including a variety of proteases and hydrolases (Saftig and Klumperman, 2009; Ballabio, 2016). The efficiency and effectiveness of lysosomal degradation of autophagosomes can be influenced by lysosome abundance, size, pH, hydrolase content, localization, and fusion (Settembre and Ballabio, 2011; Roczniak-Ferguson et al., 2012). We found previously that lysosome abundance was decreased in dystrophic muscle from mdx mice (Spaulding et al., 2018). Additionally, a large percentage of those lysosomes were observed in the extracellular space, likely affiliated with infiltrating immune cells, which would further diminish the predicted degradative power of the lysosomal pool in dystrophic muscle fibers (Spaulding et al., 2018). We also discovered that increased lysosomal abundance was associated with decreased disease severity in dystrophic muscle (Spaulding et al., 2020a). In addition, up-regulation of Src kinase may also impair autophagosome and lysosome fusion in dystrophic muscle, which would limit degradation of autophagosomal cargo (Pal et al., 2015).

Coinciding with increased interest in autophagic dysfunction in dystrophic muscle was the emergence of the D2-mdx model. The relatively novel D2-mdx mouse model was produced by backcrossing mdx mice onto the DBA background and appears to produce a more severe dystrophinopathy model compared to the conventional *mdx* mouse (Coley et al., 2016; van Putten et al., 2019). Mutations in the latent TGFβ binding protein gene (*LTBP*) cause increased fibrosis and in the *Anxa6* gene result in decreased muscle repair in the DBA background, in addition to the nonsense mutation in exon 23 of dystrophin gene, were attributed for the severity of this model compared to the mdx model (Coley et al., 2016; van Putten et al., 2019). Given the potential importance of this new model for therapeutic development (Hammers et al., 2020), the purpose of this study was to perform an initial exploration of autophagosome degradation in the D2-mdx model. We hypothesized that, similar to mdx mice, dystrophin deficiency in D2-mdx mice would cause decreased lysosomal abundance and accumulation of autophagosomes.

## Materials and Methods

### Animal Treatments

All animal procedures were approved by the Institutional Animal Care and Use Committees at the University of Montana and the University of Florida. Animals were fed *ad libitum*. Functional and histological data from the diaphragm and limb muscles from these mice has been previously published (Spaulding et al., 2019b, 2020b). Briefly, 11 month old male D2.B10-Dmdmdx/J (D2-mdx; dystrophin-deficient) and male DBA-2J (DBA; healthy control) mice were sedated to a surgical level of anesthesia, the gastrocnemius muscles and diaphragms removed, and mice were euthanized via exsanguination. Muscles were snap frozen in liquid nitrogen for subsequent analyses.

### Protein Isolation

To extract the whole homogenate, diaphragm and gastrocnemius muscles were powdered on dry ice and protein was extracted from 30 to 65 mg of muscle using a 1:10 weight to volume ratio in whole muscle extraction buffer (10 mM sodium phosphate buffer, pH 7.0, 2% SDS, 1% Halt^TM^ protease and phosphatase inhibitor single-use cocktail) using a hand-held homogenizer. Samples were centrifuged for 15 min at 1,500 × *g* and the supernatant collected in a microcentrifuge tube.

The cytosolic (CF) and lysosome-enriched fractions (LEF) were obtained from protein extracted from gastrocnemius muscles using a lysosomal isolation kit from Sigma-Aldrich (St. Louis, MO, LYSISO1) according to manufacturer instructions with minor modifications. Briefly, approximately 25 mg of powdered, frozen muscle was homogenized in a 1:4 weight to volume ratio in lysosomal extraction buffer (LEB) containing 1% protease inhibitor and centrifuged at 1,000 × *g* for 10 min. The resultant supernatant was collected and saved on ice. The pellet was resuspended in 2 volumes LEB/mg initial weight of sample, centrifuged again as above, and the supernatant was collected and pooled with the first supernatant. This process was repeated once more and the supernatant added to the pooled supernatants from the first two steps. The pooled supernatant was then centrifuged at 20,000 × *g* for 20 min. The supernatant from this step was considered the CF and was transferred to a fresh tube and stored at –80°C. The pelleted portion was considered the LEF, which was resuspended in LEB at 0.8 mL/g of original tissue weight and stored at –80°C until use. We confirmed LEF by measuring relative protein abundance of the lysosomal membrane protein, LAMP2, in both the LEF and CF using western blot (described below). LAMP2 was only detectable in the LEF.

### Western Blots

Protein concentration from whole muscle homogenate and extracted fractions were quantified using a Pierce BCA Protein Assay kit (Thermo Scientific) and diluted with Laemmli buffer (Bio-Rad), denatured at 95°C (5 min), and separated on a 4–20% precast gel (Bio-Rad; 60 V for 30 min and 120 V for 70 min). Protein from whole homogenates was diluted to 4 μg/μL and 40 μg protein was loaded into each lane. To enable comparisons between fractions, fractions were brought to an equal concentration of 0.8 μg/μL and 8 μg protein was loaded into each well on each gel. Protein was transferred to a nitrocellulose membrane at 100 V for 1 h. Following transfer, equal protein loading was confirmed by Ponceau-S staining. Ponceau-S-stained membranes were imaged and objectively quantified using AzureSpot software version 2.0.062. Total optical density did not differ between groups for any membrane though the banding pattern differed between the LEF and the CF. The membranes were rinsed in Tris buffered saline with 0.1% Tween 20 (TBST, 2 times, 2 min each) to remove Ponceau-S stain and then blocked in 5% milk (5 g non-fat dry milk in 100 mL of TBST) for 1 h. After blocking, the membranes were rinsed in TBST (2 times, 2 min each) and incubated in primary antibodies at 4°C overnight (described below), washed in TBST (3 times, 10 min each), incubated in secondary antibodies at room temperature for 1 h, washed in TBST (3 times, 10 min) and then incubated in ECL (Bio-Rad Clarity^TM^) for 6 min in the dark. Membranes were imaged using an Azure^TM^ C600 and bands were objectively measured using AzureSpot software. The lanes were identified and marked and then the bands were detected and quantified using automated band detection where possible to limit bias that may occur.

The following dilutions of antibodies from Cell Signaling (Danvers, MA) (unless otherwise noted) were used to probe membranes containing diaphragm and gastrocnemius whole muscle proteins: AMPK [Primary (P) 1:1000 TBST, Secondary (S) 1:2000 5% milk, Product number 5832S], pAMPK T172 (P 1:500 TBST, S 1:2000 TBST, 2535S), ULK1 (P 1:500 TBST, S 1:2000 TBST, 8054S), pULK1 S555 (P 1:500 TBST, S 1:2000 5% milk, 5869S), Beclin1 (P 1:500 5% milk, S 1:2000 5% milk, 3495S), pBeclin1 S91 (P 1:500 5% milk, S 1:2000 5% milk, 14717S), PI3K class III (P 1:1000 1% milk, S 1:1000 5% milk, 3358S), LAMP2 (P 1:1000 TBST, S 1:2000 5% milk, 49067S), Cathepsin B (P 1:1000 TBST, S 1:2000 5% milk, 31718S), p62 (Abcam, P 1:500 5% milk, S 1:1000 5% milk, ab56416), ATG12 (P 1:1000 TBST, S 1:2000 TBST, 4180S also used to detect ATG12-ATG5), and LC3A/B (P 1:500 5% milk, S 1:1000 5% milk, 12741S).

Several additional proteins were probed in whole muscle homogenates from diaphragm: ATG7 (P 1:1000 1% milk, S 1:1000 5% milk, 8558S), ATG16L1 (P 1:1000 1% milk, S 1:1000 5% milk, 8089S) and LAMP1 (P 1:1000 TBST, S 1:2000 5% milk, 3243S).

CF proteins from gastrocnemius muscles were probed using: AMPK [Primary (P) 1:1000 TBST, Secondary (S) 1:2000 5% milk, 5832S], pAMPK T172 (P 1:500 TBST, S 1:2000 TBST, 2535S), ULK1 (P 1:500 TBST, S 1:2000 TBST, 8054S), pULK1 S555 (P 1:500 TBST, S 1:2000 5% milk, 5869S), Beclin1 (P 1:500 5% milk, S 1:2000 5% milk, 3495S), pBeclin1 S91 (P 1:500 5% milk, S 1:2000 5% milk, 14717S), PI3K class III (P 1:1000 1% milk, S 1:1000 5% milk, 3358S), LAMP2 (P 1:1000 TBST, S 1:2000 5% milk, 49067S), Cathepsin B (P 1:1000 TBST, S 1:2000 5% milk, 31718S), p62 (Abcam, P 1:500 5% milk, S 1:1000 5% milk, ab56416), ATG12 (P 1:1000 TBST, S 1:2000 TBST, 4180S, also used to detect ATG12-ATG5), and LC3A/B (P 1:500 5% milk, S 1:1000 5% milk, 12741S).

To probe LEF we used: LAMP2 (P 1:1000 TBST, S 1:2000 TBST, 49067S), Cathepsin B (P 1:1000 TBST, S 1:2000 TBST, 31718S), p62 (Abcam, P 1:500 2.5% milk, S 1:1000 5% milk, ab56416), ATG12 (P 1:1000 2.5% milk, S 1:2000 2.5% milk, 4180S), LC3A/B (P 1:500 2.5% milk, S 1:1000 5% milk, 12741S) and ATG16L1 (P 1:1000 TBST, S 1:2000 TBST, 8089S).

### Statistics

Data were analyzed using a 2 × 2 ANOVA with main effects of disease and fraction as well as the disease x fraction in instances where probed proteins were detected in LEF and CF of both healthy and dystrophic muscle followed by a Tukey *post hoc* test. When bands were only detected in a single fraction and for whole muscle homogenate from diaphragm or gastrocnemius muscles, a Student’s unpaired *T*-test was used. All statistical analyses were performed using GraphPad Prism [version 8.2.0(272)] and significance was established at *p* < 0.05.

## Results

In previous work we established histological injury in diaphragms used in this study. Specifically, we discovered that in 11 months old D2-mdx mice fibrosis was increased by approximately 5-fold compared to healthy controls. Further, we also discovered specific tension was decreased by approximately 50% compared to healthy controls (Spaulding et al., 2020b). Similarly, in limb muscles from these D2-mdx, tetanic force and specific tension in soleus were both decreased by 30% and in EDL were decreased by 50 and 20%, respectively, compared to DBA. Fibrotic area in limb muscles increased from less than 5% in DBA to 11% in D2-mdx (Spaulding et al., 2019b).

### Markers of Autophagic Processes Differ Between Dystrophin-Deficient Diaphragms and Gastrocnemius Muscles

**FIGURE 1:**
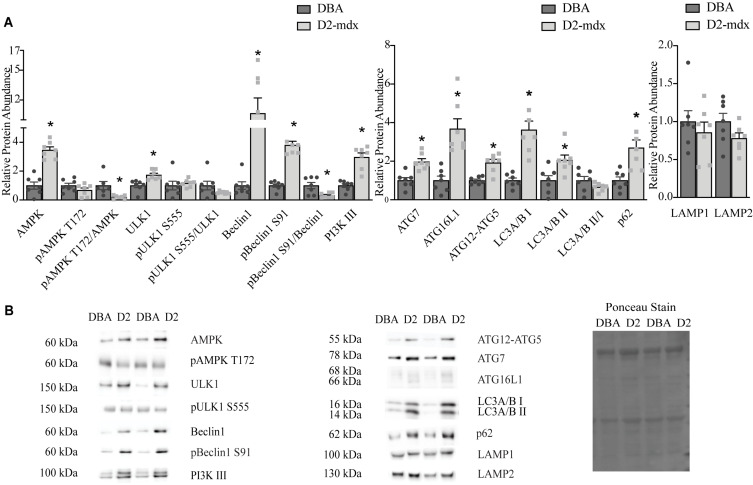
Markers of autophagy are altered in dystrophin deficient diaphragms. Western blot analysis of markers of autophagy in whole muscle extract from 11 month old DBA and D2-mdx diaphragms. **(A)** Among the activation markers, AMPK, ULK1, Beclin1, pBeclin1 S91, PI3K III were increased in D2-mdx diaphragm compared to DBA. In muscle from D2-mdx mice, the ratios pAMPK T172/AMPK and pBeclin1 S91/Beclin1 were decreased compared to muscle from DBA. The autophagosome formation markers ATG7, ATG16L1, ATG12-ATG5 were increased significantly in muscle from D2-mdx mice compared to DBA. Accumulation of autophagosomes was indicated by higher abundance of LC3 II and p62 in D2-mdx muscles compared to muscles from DBA mice. Lysosomal markers LAMP1 and LAMP2 were similar between groups. **(B)** Representative western blot images and corresponding representative Ponceau stain image used to confirm equal loading. Significance is established at *p* < 0.05. * Indicates significance between D2-mdx and DBA (*n* = 7 mice per group).

To understand how markers of autophagy were impacted by dystrophin deficiency in the D2-mdx model, we measured relative protein abundance of activation, formation, and degradation markers in diaphragms and gastrocnemius muscles from 11 month old, male animals with advanced disease. In the diaphragm, total AMPK increased 3.5-fold (*p* < 0.0001) in D2-mdx compared to DBA, pAMPK (phosphorylated at T172) was similar between groups, and the pAMPK T172/AMPK was decreased by 80% with disease (*p* < 0.05; Figure 1). Likewise, total ULK1 was increased 70% (*p* < 0.01) in diseased diaphragms, however, pULK1 (phosphorylated at S555) and pULK1 S555/ULK1 were similar between groups (Figure 1). Total Beclin1 was increased 10.7-fold (*p* < 0.0001) and pBeclin1 (phosphorylated at S91) was increased 3.8-fold (*p* < 0.0001) in D2-mdx diaphragms compared to diaphragms from DBA, resulting in a 70% (*p* < 0.05) reduction of pBeclin1 S91/total Beclin1 in dystrophic diaphragms (Figure 1). PI3 kinase class III (PI3K III) was increased by approximately 3-fold (*p* < 0.0001) in diaphragms from D2-mdx compared to diaphragms from DBA (Figure 1).

The relative abundance of autophagosome formation markers ATG7 (*p* < 0.001) and ATG12-5 (*p* < 0.001) was increased approximately 2-fold and ATG16L1 was increased by 3.7-fold (*p* < 0.001) in muscles from D2-mdx compared to DBA (Figure 1). LC3A/B I was increased by 3.6-fold (*p* < 0.0001) and the relative abundance of LC3A/B II, an autophagosome marker, was increased 2-fold (*p* < 0.05) in dystrophic muscle compared to healthy, while the LC3A/B II/I was similar between groups (Figure 1). The relative abundance of autophagic degradation marker p62 increased 2.7-fold (*p* < 0.01) in D2-mdx diaphragms compared to diaphragms from DBA mice (Figure 1). The lysosomal membrane proteins LAMP1 and LAMP2 were similar between groups (Figure 1).

**FIGURE 2:**
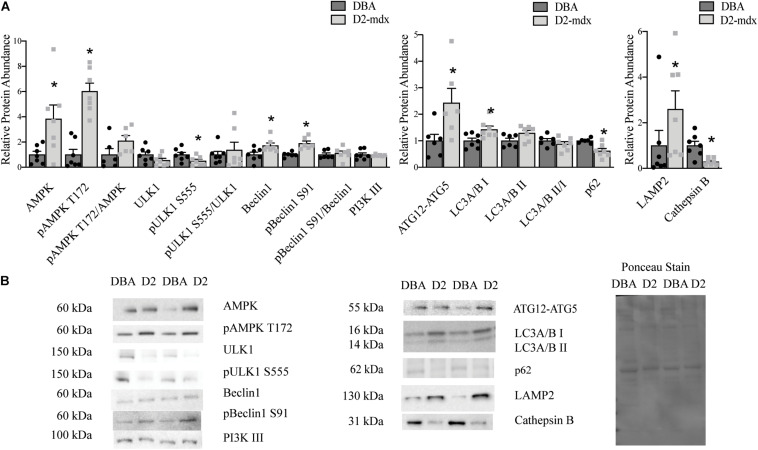
Increased autophagosome degradation in gastrocnemius from D2-mdx. Western blot analysis of autophagy markers in whole muscle extract from 11 month old D2-mdx and DBA gastrocnemius. **(A)** Among the autophagy activation markers, AMPK, pAMPK T172, Beclin1 and pBeclin1 S91 were increased in dystrophic muscle compared to DBA whereas pULK1 S555 was reduced. The autophagosome formation marker ATG12-ATG5 was increased in D2-mdx gastrocnemius muscle compared to muscle from DBA. LC3I was increased and p62 was reduced in D2-mdx gastrocnemius muscle. The lysosomal marker LAMP2 was increased and lysosomal enzyme Cathepsin B was decreased in muscle from D2-mdx compared to DBA. **(B)** Representative western blot images and corresponding Ponceau stain image. Significance is established at *p* < 0.05. * Indicates significance between D2-mdx and DBA (*n* = 7 mice per group).

To better understand the uniformity of autophagic dysfunction within different muscles from the same animals, we also probed autophagy in gastrocnemius muscles. Total AMPK was increased by 3.8-fold (*p* < 0.05), and pAMPK T172 6-fold (*p* < 0.0001), in dystrophic muscle; however, the ratio of pAMPK T172/AMPK was similar between groups (Figure 2). Total ULK1 was similar between healthy and dystrophic muscles, however, pULK1 S555 was decreased by 0.5-fold (*p* < 0.05) in D2-mdx compared to DBA and the ratio was similar between the two groups (Figure 2). Total Beclin1 was increased by 72% (*p* < 0.01) and pBeclin1 S91 was increased by 89% (*p* < 0.01) in gastrocnemius from D2-mdx mice compared to DBA and pBeclin1 S91/Beclin1 was similar between groups (Figure 2). Autophagosome formation marker ATG12-ATG5 was increased in diseased muscle by 2.5-fold (*p* < 0.05; Figure 2). LC3A/B I was increased 1.4-fold (*p* < 0.05), but the lipidated form, LC3A/B II, as well as the ratio LC3A/B II/I were similar between healthy and diseased muscles (Figure 2). p62 was reduced by 40% (*p* < 0.01) in gastrocnemius from D2-mdx compared to DBA (Figure 2). LAMP2 was increased by 2.5-fold (*p* < 0.05) in dystrophic muscles and Cathepsin B, a lysosomal cysteine protease, was reduced by approximately 75% (*p* < 0.01) in D2-mdx (Figure 2).

### Cytosolic and Lysosome Enriched Fractions

**FIGURE 3:**
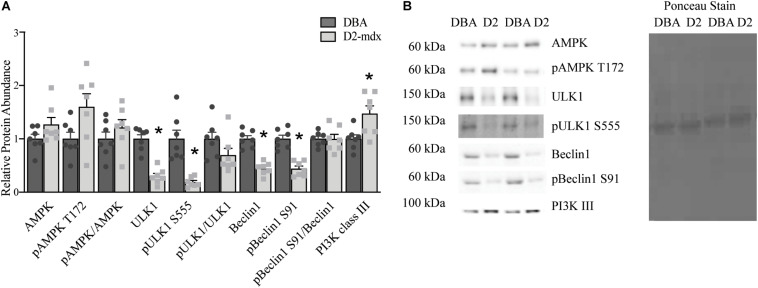
Markers of autophagic activation analyzed in a cytosolic fraction from gastrocnemius muscles. Western blot analysis of activation markers of autophagy in a cytosolic fraction from gastrocnemius muscles from 11 month old D2-mdx and DBA mice. **(A)** ULK1, pULK1 S555, Beclin1and pBeclin1 S91 were decreased, while PI3K III was elevated in D2-mdx gastrocnemius cytoplasmic fraction (CF) compared to DBA. **(B)** Representative western blot images and corresponding Ponceau stain image. Significance is established at *p* < 0.05. * Indicates significance between D2-mdx and DBA (*n* = 7 mice per group).

The findings in whole muscle extract from gastrocnemius did not support our expectation of decreased autophagosome degradation and lysosomal abundance. To better understand how autophagy may be altered by dystrophin deficiency in gastrocnemius from the D2-mdx model and to better appreciate lysosome-mediated degradation of autophagosomes, we separated the gastrocnemius into a lysosome enriched fraction (LEF) and a cytosolic fraction (CF). In the CF total AMPK, pAMPK T172, and pAMPK T172/total AMPK were similar between groups (Figure 3). Relative protein abundance of ULK1 was decreased by 70% (*p* < 0.0001) and pULK1 S555 was decreased by 80% (*p* < 0.001) in dystrophin-deficient muscle compared to healthy muscle (Figure 3). Similarly, total Beclin1 and pBeclin1 (phosphorylated at S91) were decreased by approximately 55% (*p* < 0.0001) in dystrophic muscle compared to healthy muscle (Figure 3). Because of the similar reduction in total and phosphorylated proteins, the ratios of pULK1 S555/total ULK1 and pBeclin1 S91/total Beclin1 were similar between groups (Figure 3). Relative protein abundance of PI3K III was increased by 40% (*p* < 0.05) in CF from D2-mdx compared to CF from DBA (Figure 3).

**FIGURE 4:**
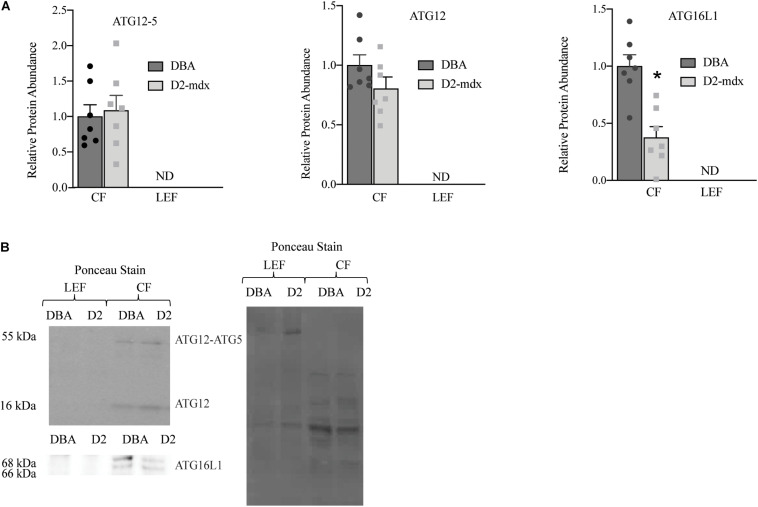
Autophagosome formation markers were detected only in cytosolic fraction from gastrocnemius muscles. Western blot analysis of ATG16L1, ATG12-ATG5, and ATG12 in cytosolic fraction of gastrocnemius muscles from 11 mo DBA and D2-mdx mice. **(A)** ATG16L1 was decreased in CF from D2-mdx compared to DBA and ATG12 and ATG12-ATG5 were similar between the groups. **(B)** Representative western blot images and corresponding Ponceau stain image. Significance is established at *p* < 0.05. * Indicates significance in D2-mdx and DBA (*n* = 7 mice per group). ND indicates not detected.

The autophagosome formation markers were only detected in the CF in healthy and dystrophic muscles. In the CF, we discovered that ATG16L1 was decreased by 63% in dystrophic muscle compared to healthy (*p* < 0.001; Figure 4). ATG12, ATG12-ATG5 complex, and the ratio between the ATG12-ATG5 complex and ATG12 were similar between groups (Figure 4).

**FIGURE 5:**
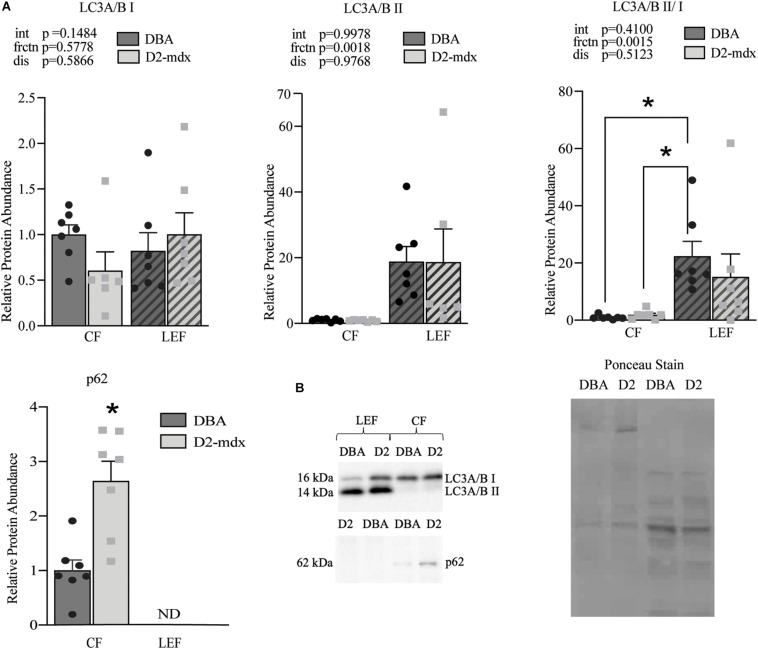
Maintenance of autophagosome degradation in fractional assessment of dystrophin-deficient gastrocnemius muscles. Western blot quantifications of LC3A/B I and II and p62 in cytosolic and lysosome-enriched fractions from D2-mdx and DBA gastrocnemius muscles. **(A)** Significant main effect due to fraction in LC3A/B II and LC3A/B II/LC3A/B I. p62 was not detected in the lysosome enriched fraction and an elevated abundance was observed in D2-mdx in the CF compared to CF in DBA mice. **(B)** Representative western blot images and corresponding ponceau stain image. Quantifications were made relative to the cytosolic fraction from the DBA group. Significance established at *p* < 0.05 (*n* = 7 mice per group). * Indicates significance between groups. *p*-values of main effects (frctn for fraction, dis for disease) and the interaction (int) are indicated, and *indicates significance between groups based on *post hoc* test linked by lines or by *T*-test (p62).

LC3A/B I and LC3A/B II were detected in both the CF and LEF (Figure 5). Relative protein abundance of LC3A/B I was similar between fractions and between groups (Figure 5). LC3A/B II was significantly decreased in CF compared to LEF but was similar as a main effect of disease (Figure 5). LC3A/B II/I was decreased in LEF as a main effect of fraction but was similar as a main effect of disease (Figure 5). p62 was only detectable in the CF, suggesting a complete degradation of p62 within lysosomes in both healthy and diseased muscles (Figure 5). In the CF, relative protein abundance of p62 was increased 2.6-fold (*p* < 0.01) in D2-mdx compared to DBA (Figure 5).

**FIGURE 6:**
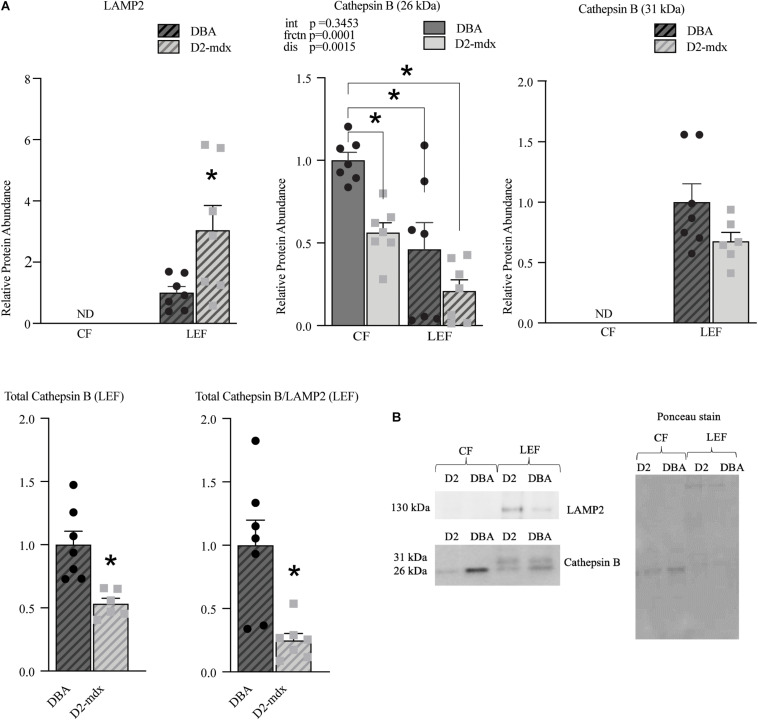
Lysosomal abundance and enzyme markers assessed in cytosolic and lysosome-enriched fractions from D2-mdx and DBA gastrocnemius muscles. Western blot protein quantifications of LAMP2, Cathepsin B (26kDa), Cathepsin B (31kDa), total Cathepsin B, and ratio total Cathepsin B/LAMP2 from cytosolic- and lysosome-enriched fractions of gastrocnemius muscles from 11 month old DBA and D2-mdx. **(A)** LAMP2 was detected only in LEF and it was increased in D2-mdx muscles compared to DBA. Within the cytosolic fraction, protein abundance of 26 kDa Cathepsin B was reduced in D2-mdx compared to DBA. The 31 kDa protein was detected only in LEF and was similar between groups. Total Cathepsin B (calculated as sum of optical densities of 31 kDa and 26 kDa) was decreased in D2-mdx LEF. Total Cathepsin B/LAMP2 was decreased in D2-mdx. **(B)** Representative western blot images and corresponding Ponceau stain image. Significance was established at *p* < 0.05. (*n* = 7 mice per group). * Indicates significance between groups by *post hoc* test (linked by lines) or by *T*-test, as appropriate. p-values of main effects (frctn for fraction, dis for disease) and the interaction (int) are indicated, ND indicates not detected.

The lysosomal membrane protein marker LAMP2 was used to confirm effective isolation of a LEF and was detected only in the LEF from DBA and D2-mdx (Figure 6). LAMP2 was increased by 3-fold (*p* < 0.05) in dystrophic muscle compared to healthy muscle (Figure 6). Cathepsin B, a lysosomal enzyme, was detected in both fractions. Of note, while Cathepsin B was detected in both fractions, the banding patterns differed substantially between the CF and the LEF, further confirming fraction isolation. Interestingly, the 31 kDa band (single chain mature Cathepsin B (Mort and Buttle, 1997; Cavallo-Medved et al., 2017) was detected only in the LEF for both DBA and D2-mdx and was similar between groups (Figure 6). The 26 kDa band, which represents the heavy chain of mature Cathepsin B protein (Mort and Buttle, 1997; Cavallo-Medved et al., 2017), was found both in the LEF as well as CF (Figure 6). There was a significant main effect of fraction such that the relative abundance was decreased by approximately 50% in LEF compared to the CF. Further, there was a main effect of disease such that D2-mdx was decreased by 50% compared to DBA (Figure 6). In the CF, the relative protein abundance was decreased by approximately 50% in dystrophic muscle compared to healthy muscle (*p* < 0.05; Figure 6). Since the banding pattern differed between the fractions, a sum of the optical density of the 26 kDa and 31 kDa bands in the LEF was considered as total Cathepsin B in this fraction. Total Cathepsin B was decreased by approximately 50% (*p* < 0.01) in dystrophic muscle compared to healthy muscle (Figure 6). To gain preliminary insight into lysosomal health we expressed Cathepsin B relative to LAMP2. Here, Cathepsin B was considered as a proxy for function since it is a lysosomal cysteine protease that contributes to degradation inside lysosomes. LAMP2 was used as an indicator of lysosomal abundance as we and others have done previously (Iwata et al., 2005; Spaulding et al., 2018; Klionsky et al., 2021). Given the known role of each protein we reasoned that the ratio of Cathepsin B to LAMP2 would provide additional insight into the health of the total lysosomal pool. We discovered that this ratio was decreased by 75% (*p* < 0.01) in dystrophic muscle compared to healthy muscle (Figure 6).

## Discussion

Duchenne muscular dystrophy is a fatal muscle degenerative disease caused by the absence of a functional dystrophin protein. Among the various cellular dysfunctions associated with DMD, upstream activation of autophagy and degradation of autophagosomes appear to be suppressed in skeletal muscles from the mdx mouse model (De Palma et al., 2012; Pauly et al., 2012; Spitali et al., 2013; Spaulding et al., 2018). Limited data from human DMD patients also indicate defective autophagy in skeletal muscle (De Palma et al., 2012; Fiacco et al., 2016). Conversely, autophagy appears to be stimulated in dystrophic hearts from rodent models, despite histopathological injury; a fact that may highlight nuanced pathology in chronically active cardiac muscle (Kang et al., 2018; Spaulding et al., 2019a). In support, cardioprotective effects were observed by treating mdx mice with rapamycin nanoparticles, and suggested amelioration of defective autophagy in dystrophic hearts (Bibee et al., 2014). Since the mdx model displays a mild disease phenotype, others have argued the more severe D2-mdx model may better recapitulate disease (Coley et al., 2016; van Putten et al., 2019) and, therefore, may more accurately predict therapeutic success in clinical trials (Hammers et al., 2020). However, how dystrophin deficiency alters autophagy in the D2-mdx model is not well-understood. We hypothesized that autophagosome degradation and lysosome abundance would be decreased in muscles from D2-mdx mice compared to muscle from DBA mice. This hypothesis was largely supported in the diaphragm as degradation of autophagosomes appeared to be suppressed. However, counter to our hypothesis, degradation of autophagosomes appeared to be stimulated in the gastrocnemius potentially due to an expansion of the lysosomal pool.

Autophagy is a multistep cellular process through which cells degrade and recycle damaged and dysfunctional cellular contents. Multiple proteins regulate activation, autophagosome formation and maturation, and degradation (Parzych and Klionsky, 2014). Canonical activation of autophagy was not robustly stimulated in diaphragm or gastrocnemius muscles, however, we identified muscle-dependent expression of some activation markers. In this experiment we also discovered elevation of pBeclin1, particularly in diaphragm, which may lead to a non-canonical activation of autophagy (Wang et al., 2009; Zhou et al., 2013). Despite the absence of upstream activation, markers of autophagosome formation and maturation were elevated in dystrophic diaphragm and gastrocnemius muscles compared to healthy muscle. These data are in good agreement with our previous work that showed elevated markers of autophagosome formation in mdx mice regardless of age (Spaulding et al., 2018).

While activation of autophagy and maturation of autophagosomes were similar between the diaphragm and gastrocnemius, degradation was substantially different. In the diaphragm, increased LC3A/B II along with increased p62 is consistent with impaired degradation of autophagosomes, while in the gastrocnemius similar LC3A/B II and decreased p62 is supportive of increased degradation of autophagosomes (Ichimura and Komatsu, 2010). Maintenance of autophagosome degradation in limb muscle is associated with attenuated functional decline in limb muscles (soleus and EDL) compared to diaphragms all from these animals (Spaulding et al., 2019b, 2020b). The mechanism underlying this fundamental difference is beyond the scope of this investigation, though speculatively may be influenced by the different activation patterns of these muscles, exposure to different forces, their differing fiber types, and/or differing metabolic profiles (Silverman and Atwood, 1980; Parry and Parslow, 1981; Petrof et al., 1993a; Zardini and Parry, 1994; Augusto et al., 2004), compounded by different rates of disease progression (Hammers et al., 2020). While muscle-dependent differences in autophagy have been previously reported, to our knowledge, the mechanism underlying these differences has not been fully elucidated (Mizushima et al., 2004; Raben et al., 2008; Ogata et al., 2010; Mofarrahi et al., 2013; Fernando et al., 2020).

In a previous experiment we discovered that lysosome abundance was decreased in dystrophic diaphragms with advanced disease (Spaulding et al., 2018). Independently, we also reported that stimulation of lysosomal biogenesis was associated with increased degradation of autophagosomes and decreased disease severity (Spaulding et al., 2020a). Collectively, these data indicate that impaired degradation of autophagosomes is due, at least in part, to decreased lysosomal content in dystrophic muscle from mdx mice. In this investigation using the D2-mdx model we found that in diaphragms with advanced disease lysosomal content was similar between groups and we identify this as a potential difference between these models, though acknowledge the differences in age of mice in our previous work and the work contained herein. Importantly though, while mice were of different ages (e.g., 17 mo old mdx mice and 11 mo old D2-mdx mice) muscles from both experiments had profound disease-related injury. Conversely, in D2-mdx gastrocnemius muscles, lysosomal abundance was elevated, which may allow maintenance of degradative power. Speculatively, decreased Cathepsin B may be suggestive of impaired lysosomal function. Hence, a larger pool of less functional lysosomes may be necessary to maintain total lysosomal function and degradation of autophagosomes; a point emphasized by findings in associated LEFs. In the gastrocnemius, increased degradation of autophagosomes contradicts findings from mdx mice as well as human patients, which indicate autophagosome accumulation in dystrophic muscles (De Palma et al., 2012; Pauly et al., 2012; Spitali et al., 2013; Spaulding et al., 2018, 2020a).

As maintained, or even increased, degradation of autophagosomes was unexpected in dystrophin-deficient muscle, to better understand degradation of autophagosomes in gastrocnemius muscles, the autophagy pathway was further assessed by comparing changes within CF and LEF. Consistent with findings from whole homogenate, upstream activation of autophagy and initiation of autophagosome formation did not appear to be robustly stimulated in the CF. Interestingly, ATG12-ATG5, ATG12, and ATG16L1 were found only in the CF, supporting previous discoveries indicating dissociation of this complex during autophagosome maturation (Mizushima et al., 2001; Kuma et al., 2002). That p62 was found in the CF, but not the LEF, along with similar LC3A/B II in the LEF, indicates functional lysosomal degradation of autophagosomes in dystrophin-deficient (and healthy) gastrocnemius muscles. Detection of LC3A/B I in the LEF is congruent with previous findings (Yang et al., 2011). Consistent with our observations from whole homogenates, in LEF lysosome abundance was increased in dystrophic muscle compared to healthy muscle. Importantly, and supportive of isolation of an LEF, in healthy and dystrophin-deficient muscle lysosomes were only detected in the LEF. Given these outcomes it is reasonable to suggest the increased lysosomal pool, at least in part, maintained degradation of autophagosomes in dystrophic gastrocnemius muscles raising the possibility that a larger lysosomal pool was, indeed, necessary to achieve the same degree of degradation as in healthy muscles. To gain preliminary insight into lysosomal health or function we expressed total Cathepsin B abundance relative to lysosomal abundance and discovered that lysosomes from dystrophic muscle have only 25% of total Cathepsin B compared to lysosomes from healthy muscle. These data raise the possibility of impaired lysosomal function in dystrophic muscle, which is mitigated, at least in part, by a larger lysosomal pool.

There are several limitations that should be considered when interpreting data presented here in. Though cellular fractionation provides an excellent tool for lysosomal isolation, presence of some non-lysosomal components is possible (Oberle et al., 2010). This was our rationale for specific use of the term LEF, rather than lysosomal fraction. In addition, though whole muscle is composed of heterogeneous cell types, including muscle fibers, satellite cells, immune cells, endothelial cells, fibroblasts etc., the vast majority is comprised of muscle fibers (Rubenstein et al., 2020). Given the incredible disparity in muscle fiber volume, mass, and nuclei relative to other cell types it seems unlikely that interpretation of present data would be significantly impacted by changes in these other cell types. Finally, we acknowledge some differences in outcomes measured in whole homogenate and measured in CF. These differences are likely due to differences in the retained components in the whole homogenate and the CF and may also point to disease-related impacts on protein localization or interaction (Strappazzon et al., 2011; Seibenhener et al., 2013; Liu et al., 2016; Lan et al., 2018).

## Conclusion

We discovered that markers of autophagy differ between diaphragms and gastrocnemius muscles from D2-mdx mice. Most substantially, in diaphragms, degradation of autophagosomes appears blunted, however, in gastrocnemius muscle degradation of autophagosomes appears to be functional due, at least in part, to an increased lysosomal pool. This notion was further supported by deeper investigation using a LEF, which also raised the possibility of a larger lysosomal pool coupled with impaired lysosomal function. In addition to providing novel insight regarding autophagic degradation in an emerging mouse model, these data indicate the need for careful consideration of disease model and muscle of interest in future studies of autophagy.

## Data Availability Statement

The original contributions presented in the study are included in the article/supplementary material, further inquiries can be directed to the corresponding author.

## Ethics Statement

The animal study was reviewed and approved by the Institutional Animal Care and Use Committees at the University of Montana and the University of Florida.

## Author Contributions

JS and JQ designed the animal experiment. JS, HS, SK, and MH conceived the idea for this experiment and interpreted the data. Tissue and data collection, and data analysis were performed by SK, HS, JQ, and TQ. JS and SK drafted the manuscript. All authors contributed in manuscript revision and editing.

## Conflict of Interest

JS and MH are founders of Extrave Bioscience, LLC. This work does not have any obvious connection to goals of Extrave Bioscience, LLC. The remaining authors declare that the research was conducted in the absence of any commercial or financial relationships that could be construed as a potential conflict of interest.
